# Overconfident, but angry at least. AI-Based investigation of facial emotional expressions and self-assessment bias in human adults

**DOI:** 10.1186/s40359-025-02590-7

**Published:** 2025-03-10

**Authors:** Roland Kasek, Enikő Sepsi, Imre Lázár

**Affiliations:** 1 Institute of Behavioural Sciences, Semmelweis University, Budapest, Hungary; 2 Institute of Arts Studies and General Humanities, Károli Gáspár University of the Reformed Church, Budapest, Hungary

**Keywords:** Facial emotional expression, Self-assessment bias, Nonverbal communication, Metacognition, Artificial intelligence

## Abstract

Metacognition and facial emotional expressions both play a major role in human social interactions [1, 2] as inner narrative and primary communicational display, and both are limited by self-monitoring, control and their interaction with personal and social reference frames. The study aims to investigate how metacognitive abilities relate to facial emotional expressions, as the inner narrative of a subject might project subconsciously and primes facial emotional expressions in a non-social setting. Subjects were presented online to a set of digitalised short-term memory tasks and attended a screening of artistic and artificial stimuli, where their facial emotional expressions were recorded and analyzed by artificial intelligence. Results show self-assessment bias in association with emotional expressivity – neutrality, saturation, transparency – and the display of anger and hostility as an individually specific trait expressed at modality-dependent degrees. Our results indicate that self-assessment bias interplays in subconscious communication – the expression, control and recognition of facial emotions, especially – with empathetic skills and manipulation.

**Supplementary Information:**

The online version contains supplementary material available at 10.1186/s40359-025-02590-7.

## Introduction

Nonverbal communication holds significant importance in shaping interpersonal relationships, serving as a channel for expressing emotions and conveying information above content, it complements and alters primary meanings. Human emotion perception and recognition are automatic, enhanced by both organic (mirror neurons) and functional abilities (empathy).

Observation of facial muscles movements has been shown in many studies to be sufficient in itself to assess the emotional state of a subject [3–6] enabled by distinct neuronal pathways [7], and in specific cases to show universal patterns across species and cultures [8]. For the basic emotions of joy, surprise, anger, disgust, sadness, fear, it is generally accepted that they can be reliably described by identical faciomuscular movement configurations and that they have individual, social and other meanings beyond the expression of emotion [9]. 

Although the recent academic debate argues whether or not emotions are directly linked to facial expressions and challenges the status quo that they are universally recognized as summarized by Heaven [10], Cowen et al. [11] suggests a high-dimensional framework to map the phenomena of human emotional expression and experience.

### Artificial intelligence and emotion recognition

Recent studies conclude that while facial expressions are seemingly universal with a culturally dependent saturation of meaning, the underlying affective states rarely correlate with emotions that, in this sense, consist of spontaneous neurobiological changes and their cognitive evaluation, leading to cognition-dependent activation or inhibition of specific conscious and instinctive responses [12, 13].

With the development of imaging and image analysis technologies, it has also become possible to code facial expressions and recognize emotions based on categorical [5] or continuous [14] models using artificial neural networks and machine learning in real time from recordings to use the resulting data for analysis, primarily for marketing purposes. [eg. 15,16] However, Automatic Facial Coding (AFC) systems developed to understand and influence consumers’ emotions in line with business purposes, are also ideally suited for psychological assessments, taking their limitations and potential into account and benefit both the research and the development [17].

The discrepancies of systematically manipulated content - e.g. the role of modalities, influence of artistic effects - in individual and social situations can be directly investigated when using high-quality videos recorded under laboratory conditions [18]. At the same time, when complemented with psychophysiological and psychometric measurement tools, AFC systems allow for sensitive data acquisition and – compared to traditional methods based on typically self-reported phenomenological interpretations – enables highly accurate statistical analysis, especially in the context of implicit process diagnostics [19].

A further advantage of video-based emotion recognition is that it can be analyzed with a machine learning and artificial intelligence-enabled system, based on the judgement of emotion recognition experts – developed in time-consuming and expensive recruitment procedures – cross-referenced with relevant investigations, so that the human error can be eliminated in the analysis, resulting in AFCs to outperform humans [20].

### Evolutionary background

Humans, compared to apes [21], exhibit reduced short-term visual memory in urban settings but excel in distinct cortical functioning e.g. long-term memory, imagination, and intentionality in deceit detection. The increased neocortex supports a reduced energy consumption, enhancing cognitive capacity for logical thinking and intention attribution in long-term mating strategy [22].

Despite inherent inaccuracies [23], imagination plays a vital role in creating adaptive strategies for varying situations and stakes, hence assists risk assessment, meaning constant awareness and ability to mentally manipulate any and all information about the potential partner in order to reinforce or inhibit affiliation [24].

### Facial emotional expression in social interactions

According to Ekman and Friesen [25], lying is nothing more than a dissonance of cognition and emotion that leads to nonverbal leakage along channels. The modality constraint and the separation of channels greatly affect the observer’s picture of the observed person’s affective-cognitive coherence - even without the content [26].

Analyzing facial expression, as a pivotal channel in human interactions [27], in terms of cognitive functions it is clear that it engages visual short-term and semantic visual memories associated with vision and its processing [28], just as much as the decoding of body language [29].

Human cognition extends beyond processing ‘what is’ to also consider ‘what could be’ in nonverbal communication as well, particularly deception detection, which relies on brief visual cues, with the ‘presentation’ time of micro-expressions being as short as 1/5 − 1/25 milliseconds [25, 30]. The challenge lies in the detection of the emotional leakage that manifests in micro-expressions and contradicts the primary message. Detecting nonverbal leakage is complex, compounded by its atypical appearance. In human social interactions, distorted truths foster justifications, confirming suspicions and breeding stronger illusions to resolve cognitive dissonance.

While cognitive biases and stereotypes can be useful in everyday life, peer influence also puts evolutionary pressure on self-deception as an adaptive strategy [31], they are activated uncontrollably when the right risk (time pressure, stakes, danger, emotional context) is present, and it is almost impossible to ignore them.

Just like in Flavell’s [32] subjects, self-awareness [33] is key to performance – from fast and accurate perception to being able to be simultaneously aware of a situation and its possible outcomes – and especially in communication, probabilistic prediction of intentions and successful manipulation of others – and a capacity that allows these simultaneous processes to perform efficiently – allows one to time the use of resources optimally, and being aware of both limitations and potentials. Awareness relieves cognitive capacity of unnecessary burdens, and volitional inhibition can be applied to types of nonverbal leakage that are more difficult to detect, while those that are easier to assess lead to more accurate – or, defined by the inner speech or narrative, self-assuring – findings.

## Aim

In social interactions, intentional masking and display of emotional states lead to the appearance of micro-expressions and empathy-driven mimicry, therefore, we have hypothesised that the defining inner narrative enabled by self-awareness – based on self-confidence and personality – of a subject might project subconsciously and primes facial emotional expressions. To observe such phenomena and let faces serve as a purely inner-state dependent display, subjects were prevented from maintaining interaction during data collection, hence allowing honest – or at least less deceitful – reactions to stimuli.

While laboratory settings offer a sterile context to capture what might really be happening, they also miss out on revealing the real-life characteristics of social interactions.

Our goal was to develop a setting that allows more space for the observation of the life-like approach to facial emotional expression using artistic and artificial experience as tools to understand the differences in emotional expressions and experiences between man-made and machine generated stimuli.

As activation and inhibition of expressing emotions are motivated and enabled by individually different interplay of cognitive and affective factors, we have also hypothesized that the removal of direct social contact will promote the temporal and qualitative extension of facio-muscular activation and therefore reveal otherwise voluntarily or subconsciously hidden characteristics of the associations between self-confidence, personality traits and facial emotional expressions.

## Method

Subjects were presented online on their own devices to a set of digitalised short-term memory tasks [34] that measure cognitive capacity and metacognitive abilities simultaneously. In the second phase of the experiment a subgroup of previously tested adults based on availability and willingness attended a screening of artificial audio, human audio and video stimuli, where their actions were recorded for further facial emotional expression analysis with Noldus FaceReader 8.1 as described in the graphical abstract (Appendix GA).

### Participants

The study investigates a sample of 35 mentally healthy native Hungarian adult subjects – 14 men (age = 28–51 years; M = 35.1 years) and 21 women (age = 19–48; M = 30.8 years) – who were randomly selected from a previously psychometrically tested larger population of volunteers recruited at the university and by social media based on availability and willingness. Before participating, subjects anonymously stated that they are mentally healthy adults and consented to the collection and use of their data.

### Laboratory equipment and setting

Close-up studio cameras were set up in front of the subjects while they listened to the three literary excerpts in Hungarian with a total duration of 16 min interpreted by a professional actor in order: first with actor-performed audio (sound only), then with actor-performed experience (sound and image), and finally with artificially generated audio (sound only). After the first screening the order of stimuli was shuffled to avoid artefacts that may arise from order effect confound, despite the fact that we were only going to investigate modality dependent changes and individually specific emotion expressions.

### Apparatus

#### Performance tests

The test software includes four memory tasks (span of 3 to 9) of increasing complexity. The basic task (Task 1) is a digitalised version of the Corsi Block Tapping Test [35], requiring the correct sequential localization of sequentially presented homogeneous stimulus material (dots), while the second task (Task 2) requires the spatial recall of simultaneously presented numbers in ascending order [36]. The third task (Task 3) is a repetition of the second with a self-paced presentation time [32]. The fourth task (Task 4) requires spatial-numerical interference inhibition and correct spatial recall [34]. At the end of the tasks, participants intuitively rate their own performance relative to their peers on a Likert-scale of 6 [37].

#### Facial action coding with artificial intelligence

The FaceReader by Noldus uses an artificial neural network to classify emotional expressions, which yields data such as basic expressions, individual expressions, head orientation, gaze direction, personality characteristics, valence and arousal, as well as heart rate and heart rate variability with frame-by-frame resolution limited by the analyzed video material’s frame rate and outputs data with milliseconds accuracy. By default, the diagnostic software measures the expression of the six basic emotions (joy, fear, anger, sadness, surprise, disgust) as a percentage and the temporal expression of the most prominent emotion along reaction time. A validation study conducted by Stöckli et al. [38] found that FaceReader 6 performed the best of the major emotion classification software available at the time, with an average accuracy of 88%. Another study done by Lewinski, den Uyl and Butler [19] states that FaceReader correctly recognized 88% of expressions on average in the WSEFEP and ADFES pictures, whereas human participants only recognized 85%, and outperforms humans 90 to 59% when it comes to neutral faces [20]. FaceReader 8.0 reached a higher test-retest reliability than human coders [39], and the since improved version of FaceReader - version 8.1 was used in this study - achieved an even higher score of 96% in emotional recognition according to the developer, so we can at least assume it is as good as or even better than human coders and valid in minimum 88% of the cases.

#### Statistical analysis

Descriptive statistics, Kruskal-Wallis tests with Dwass-Steel-Critchlow-Fligner pairwise comparisons and Brunner-Munzel tests – the most reliable nonparametric procedure for relatively small sample comparisons [40] – were conducted using jamovi 2.3 [41] for the software’s unique ability to perform the latter. The threshold for statistical significance was set at *p* <.05.

### Variables

#### Performance and self-assessment

Memory performance (MP) was measured simply by sum of hits across all tasks excluding effortlessly acquirable – first and last – hits out of the accumulation. Based on hits accumulated across the four tasks, results were recorded test by test as baseline for the post-task self-assessment Likert-scale of 1–6, that was compared to actual, 1–6 scaled performance and subtracted and averaged across T1-T4 into self-assessment bias index (SAB), allowing the grouping of subjects by bias polarity–lower or higher than 0 – (P_SAB_) and accurate or biased self-confidence (A_SAB_).

#### Facial action coding with artificial intelligence

Facial emotional expressions were recorded and analysed throughout the entire screening time with a chosen analysis frame rate of 30 frames per second resolution (Borges et al., 2019) – a unique ability of the artificial intelligence – into cumulated percentage values by basic (happy, surprised, sad, scared, disgusted, angry), other and neutral emotion category and each modality (‘HV Angry 28.36’ for the total percentage of anger expressed during each Human Video setting), then averaged into a modality-independent facial expression index (‘A_E_ Angry 12.31’).

Based on our preliminary exploration of the raw recordings and the collected data, we have identified the need to develop emotional expression ratios as new variables to be able to compare the characteristics across different modalities. Emotional Saturation (S_E_) shows the ratio of ‘dominant’ to ‘all other’ emotions, to explore the subject’s emotional span. Emotional Transparency (T_E_) represents the ratio of ‘basic six’ to ‘other’ emotions and reveals how difficult might be to decode the social target’s emotional state just by registering their facial expressions.

#### Emotion experience and personality

Since emotions and personality traits are interrelated [eg. 42,43], based on therapeutic considerations [44] we have grouped basic emotions into three personality categories – Extroversion (happy and surprised), Neuroticism (sad and scared) and Hostility (disgusted and angry) by Izard’s theoretical framework and results [45] – to be able to analyse the associations of self-confidence and personality dimensions as defining factors of how subjects empathize, experience and express emotions.

## Results

### Gender differences

Brunner-Munzel testing confirmed no evidence of gender differences (Table 1) in our sample regarding age (Age), memory performance (MP), self-assessment bias (SAB), accuracy (A_SAB_) and polarity (P_SAB_).

**Table 1:** Brunner-Munzel tests confirms no evidence of differences between basic variables (Age, memory performance, Self-Assessment bias, accuracy and Polarity) and biological gender (Sex)

	Statistic	df	*p*	Relative effect
AGE	-1.559	31.8	0.129	0.350
MP	-0.182	29.9	0.857	0.481
SAB	-0.441	28.9	0.662	0.459
A_SAB_	0.406	24.3	0.688	0.524
P_SAB_	-0.538	27.9	0.595	0.452

Note. H_a_*P* ^(1 < 2) + ½*P* ^(1 = 2) ≠ ½

### Modalities

Compared to audio presentation (sound only), the full artistic experience (sound and video) seemingly rather dimmed than enhanced emotional expressions. Contrary to our expectations, Kruskal-Wallis testing of emotional expressions by modalities – Human Audio, Human Video, Artificial Audio and their modality-independent Average – confirmed no evidence of such phenomena (Table 2), even in DSCF pairwise comparisons (Appendix Table 1 A).

**Table 2:** Kruskal-Wallis tests confirms no evidence of differences between each modality (HA, HV, AA) and their average (A_E_)

Emotion	χ²	df	*p*	ε²
Neutral	0.740	3	0.864	0.00532
Happy	0.741	3	0.863	0.00533
Surprised	2.249	3	0.522	0.01618
Sad	0.608	3	0.895	0.00437
Scared	2.015	3	0.569	0.01449
Disgusted	0.540	3	0.910	0.00388
Angry	3.074	3	0.380	0.02212
Other	5.107	3	0.164	0.03674
S_E_	1.801	3	0.615	0.01296
T_E_	0.343	3	0.952	0.00247
Extroversion	1.473	3	0.689	0.01060
Neuroticism	0.530	3	0.912	0.00382
Hostility	2.511	3	0.473	0.01807

Note. * *p* <.05 ** *p* <.01, *** *p* <.001

### Self-assessment bias and emotions

General analysis of the sample revealed that self-assessment bias is moderately associated with the expression of anger (χ²= 7.3736, df = 2, *p* =.25, ε²=0.21687) and hostile tendency (χ²= 7.4567, df = 2, *p* =.24, ε²=0.21931). Surprisingly and quite contrary to our expectations based on a previous phenomenological approach [46], the difference between the expressions of other basic emotions were not significant (Table 3).

**Table 3:** Kruskal-Wallis tests confirms evidence of differences between self-assessment bias (SAB) and the average of facial emotional expressions (A_E_ angry, A_E_ Hostility)

Emotion	χ²	df	*p*	ε²
Neutral	3.7451	2	0.154	0.11015
Happy	2.3276	2	0.312	0.06846
Surprised	0.0597	2	0.971	0.00176
Sad	2.0917	2	0.351	0.06152
Scared	1.5000	2	0.472	0.04412
Disgusted	2.6545	2	0.265	0.07807
**Angry**	**7.3736**	**2**	0.025*	**0.21687**
Other	5.3169	2	0.070	0.15638
S_E_	3.5787	2	0.167	0.10526
T_E_	2.7109	2	0.258	0.07973
Extroversion	1.3546	2	0.508	0.03984
Neuroticism	2.0917	2	0.351	0.06152
**Hostility**	**7.4567**	**2**	0.024*	**0.21931**

Note. * *p* <.05, ** *p* <.01, *** *p* <.001

#### Self-assessment accuracy

Categorical grouping and Brunner-Munzel testing (Table 4) of our sample by accurate or biased self-assessment (A_SAB_) confirmed evidences that Neutrality (BM = 2.97, df = 5.58, *p* =.014, RE = 0.798) as lack the of detectable expressions and Transparency (BM = 2.33, df = 26.01, *p* =.014, RE = 0.702) as the incidence of basic emotional expressions was strongly higher, while Saturation (BM=-2.62, df = 9.08, *p* =.005, RE = 0.210) as the variety of emotional experiences was moderately lower in the biased group.

Exploring the differences between modalities, the artificial audio (AA) setting emerged both in emotional Neutrality (BM = 4.03, df = 5.50, *p* =.004, RE = 0.847) and Transparency (BM = 4.11, df = 12.28, *p* <.001, RE = 0.819) in the biased group with a strong relative effect, while the human audio (HA) setting elicited moderately Saturated expressions (BM=-2.72, df = 14.51, *p* =.008, RE = 0.258) in the accurate group.

**Table 4:** Brunner-Munzel tests confirms evidence of differences by self-assessment bias accuracy (A_SAB_) and emotional neutrality, emotional saturation (S_E_) and emotional transparency (T_E_) across modalities and their average (A_E_)

Emotion	Modality	Statistic	df	*p*	Relative effect
Neutral	HA	1.88	8.96	0.047*	0.694
	HV	2.87	4.20	0.021*	0.815
	**AA**	**4.03**	**5.50**	0.004**	**0.847**
	A_E_	2.97	5.58	0.014*	0.798
**S**_**E**_	**HA**	**-2.72**	**14.51**	0.008**	**0.258**
	HV	-1.87	4.27	0.065	0.258
	AA	-2.62	7.56	0.016*	0.242
	**A**_**E**_	**-3.26**	**9.08**	0.005**	**0.210**
T_E_	HA	1.33	3.96	0.128	0.694
	HV	1.14	4.18	0.158	0.665
	**AA**	**4.11**	**12.28**	< 0.001***	**0.819**
	A_E_	2.33	26.01	0.014*	0.702

Note. H_a_*P* ^(1 < 2) + ½*P* ^(1 = 2) > ½; * *p* <.05, ** *p* <.01, *** *p* <.001

#### Self-assessment bias Polarity

Brunner-Munzel tests revealed (Table 5) relatively strong associations of positive bias polarity (P_SAB_) and Hostility across modalities and their Average (BM = 2.91, df = 33.0, *p* =.003, RE = 0.732), as a result of strong synergetic tendencies of Disgust (BM = 1.74, df = 27.0, *p* =.047, RE = 0.608) and Anger (BM = 2.35, df = 27.0, *p* =.013, RE = 0.670).

**Table 5:** Brunner-Munzel tests confirms evidence of differences by self-assessment bias Polarity (P_SAB_) and disgust, anger and hostility across modalities and their average (A_E_)

Emotion	Modality	Statistic	df	*p*	Relative effect
**Disgusted**	**HA**	**1.74**	**27.0**	0.047*	**0.608**
	HV	1.67	29.0	0.053	0.605
	AA	0.97	30.1	0.171	0.552
	A_E_	1.67	29.0	0.053	0.605
**Angry**	**HA**	**2.35**	**27.0**	0.013*	**0.670**
	HV	1.67	29.0	0.053	0.605
	AA	1.46	25.0	0.078	0.585
	**A**_**E**_	**2.40**	**31.9**	0.011*	**0.681**
**Hostility**	**HA**	**3.38**	**31.3**	< 0.001***	**0.755**
	**HV**	**1.93**	**32.9**	0.031*	**0.647**
	**AA**	**1.82**	**30.7**	0.039*	**0.632**
	**A**_**E**_	**2.91**	**33.0**	0.003**	**0.732**

Note. H_a_*P* ^(1 < 2) + ½*P* ^(1 = 2) > ½; * *p* <.05, ** *p* <.01, *** *p* <.001

### Summary

Altogether, the subjects of the observed sample reacted typically with neutral expressions to the presented stimuli, which can be explained by the reduced attentional capacity and the resulting lower receptivity and empathy due to the fear of the epidemic and the stress arising from the restrictions, as well as by the laboratory situation itself.

It is also a surprising result that despite the lack of direct evidence of emotional experiences between modalities, we have found strong but modality-dependent associations with self-assessment bias and emotional expressions, which leads to several conclusions:

- That the actor’s predominantly negative-neutral facial expressions influence the recipient’s facial expressions in terms of affective transfer; and.
- That the emotional intensity of the audio-only experience is higher because this form presumably allows more room for imagination, hence reserves cognitive processing power to the detriment of the control of facial expressions.

The artificial sound, on the other hand, has often resulted both in temporary serenity in case of negative emotions and in enhanced positive emotions, most probably because of its comic intonation and inconsistent emphasis which were violating natural patterns, resulting in relatively strong associations between self-assessment accuracy, emotional transparency and neutrality. Accurate self-assessment is associated with lower facial emotional control (lower Neutrality), higher emotional Saturation and lower Transparency, compared to any sign of bias, while Anger and Hostility are typical in the overconfident group.

## Discussion

### Self-reflection and facial emotional expression

The aim of the study was to investigate how cognitive capacity and metacognitive abilities relate to facial emotional expressions. The short-term memory tasks were essential to measure an objective output in a non-familiar situation, on which subjects were able to intuitively reflect on and estimate their performance on a scale of 1–6 compared to peers. Processing these objective and subjective factors gained us access to the individually attributive degree of self-assessment bias, which allowed the exploration of the subjects’ inner narrative without the typical influencing factors of self-reported results. To let faces serve as a purely inner-state dependent display, subjects were prevented to maintain interaction during the screening, hence allowing unfiltered – or at least less voluntary – facial emotional reactions to stimuli.

As we have hypothesised, the defining inner narrative of a subject projects subconsciously towards peers and influences facial emotional expressions. The intensity of facial emotional expression depends on whether and to what extent the subject is organically and functionally able and willing to communicate emotional state to others in a given situation. Cognitive capacity directly determines the ability of self-control – associated with the approach or inhibition of certain behaviours – and metacognitive ability which together influence self-awareness, social interactions, and are particularly relevant in the case of intentional expression or repression of facial emotions. However, in the applied laboratory setting none of this mattered: subjects were practically prevented interaction, and their purely receiving role allowed us to observe facio-muscular reactions without their normally active facial expression filters.

Happiness, sadness and surprise appeared on the faces regardless of the array and magnitude of self-assessment bias, leading us to a conclusion that their inner narrative aligned with past experiences and primes expressions by eliciting sympathy and support in others for the results – whether they lag behind promises or meet expectations – might be independent of self-confidence. Hostile – disgust and mainly angeremotional expressions, on the contrary, stem in overconfidence and mitigates the expected harm that might be done by others for the results are thought to fall short of requirements, providing a higher ground in a predicted conflict on a ‘best defense is – attack’ basis and as a constructive, reparative response [47]. Our finding indicates that further research may explore how overconfidence exactly relates to inner speech, the expression of anger and hostility, and whether and how these results might serve therapeutic aspects.

Neutrality and happiness were detected in cases of congruent self-reflection, independent of cognitive capacity but related to gender disparities. Male subjects of minimally biased or precise self-assessment seemingly enjoyed the screening more than females, which can either be attributed to.

- The male actor’s appearance or the play itself,
- The mood and topics of the literary excerpts, or.
- Simply the fact they were able to relax and focus on the experience of having a normally peaceful inner narrative due to their accurate self-reflection and social reference frame.

In conclusion, self-assessment bias seemingly related to how subjects manipulate their environment, and depending on their projected expectations based on past experiences, self-confidence and personality, their expression reflected on how they want others to see them: hostile, friendly or lost.

### Nonverbal communication in solitary

The costs and benefits of expressing or repressing emotional states are purely situational and depend on capacity and motivation. Whilst in social interactions sympathy, empathy – a special form of metacognition, being aware and in control of feelings – and intention define verbal-nonverbal congruency, inhibition of emotional leakage becomes futile for subjects who are experiencing temporary social deprivation or isolation.

The reception of artistic performance as a one-way communication triggered more intense emotional reactions when imagination reserved cognitive capacity, while the appearance of the actor saturated the experience resulting in similar, but decreased amplitude of facial expressions. Observing such phenomena allows us to conclude that artistic audio-visual experience might develop empathy and empathetic skills by solitary exploration of affective changes which improve self-awareness by practicing adaptation to another person without the consequences of the attempt.

In conclusion, the removal of direct social contact promotes the temporal and qualitative extension of facial emotional expressions and therefore linked to cognitive capacity, metacognitive abilities and personality traits.

## Constraints and limitations

Although the relatively small sample size, the self-reported mental health status and known effects of the pandemic – and its due restrictions – on cognitive abilities [48, 49] prevent us from directly generalize these findings to the majority of the population, our results indicate that further investigation of the yet revealed pattern would be beneficiary in the development of diagnostic tools and therapeutic interventions.

The experiments were carried out between May and June 2020, after months of obligatory use of surgical masks, social deprivation, existential threat and uncertainty which altogether might have altered emotional experience and expression due to known increase in mental illnesses and disorders [50, 51]. It is important to underline that recent findings suggest that isolation and physical distancing itself - which has been misinterpreted by mainstream media as social distancing - may also be responsible for functional uncertainty, as it is associated with neuropsychological and neurobiological changes in early childhood and with age [52], and therefore may alter nonverbal communication patterns.

Further and better funded investigation involving artificial intelligence that enables temporal data processing – e.g.: emotional Saturation and Transparency – in a larger sample may also be required to determine whether and how metacognitive abilities interplay in the expression of facial emotions and recognition, especially in relation with psychophysiological correlates – e.g. heart rate variability, galvanic skin resistance – of emotion production, empathetic skills and manipulation.

## Electronic supplementary material

Below is the link to the electronic supplementary material.

Supplementary Material 1

Supplementary Material 2

## Data Availability

The data presented in this study are available upon request from the corresponding author for legal agreements and ethical reasons.
