# Supplementary figures and images for: Overconfident, but angry at least. AI-Based investigation of facial emotional expressions and self-assessment bias in human adults
Source: BMC Psychol. 2025 Mar 10;13:223. doi: 10.1186/s40359-025-02590-7 (PMC11895137; doi:10.1186/s40359-025-02590-7)

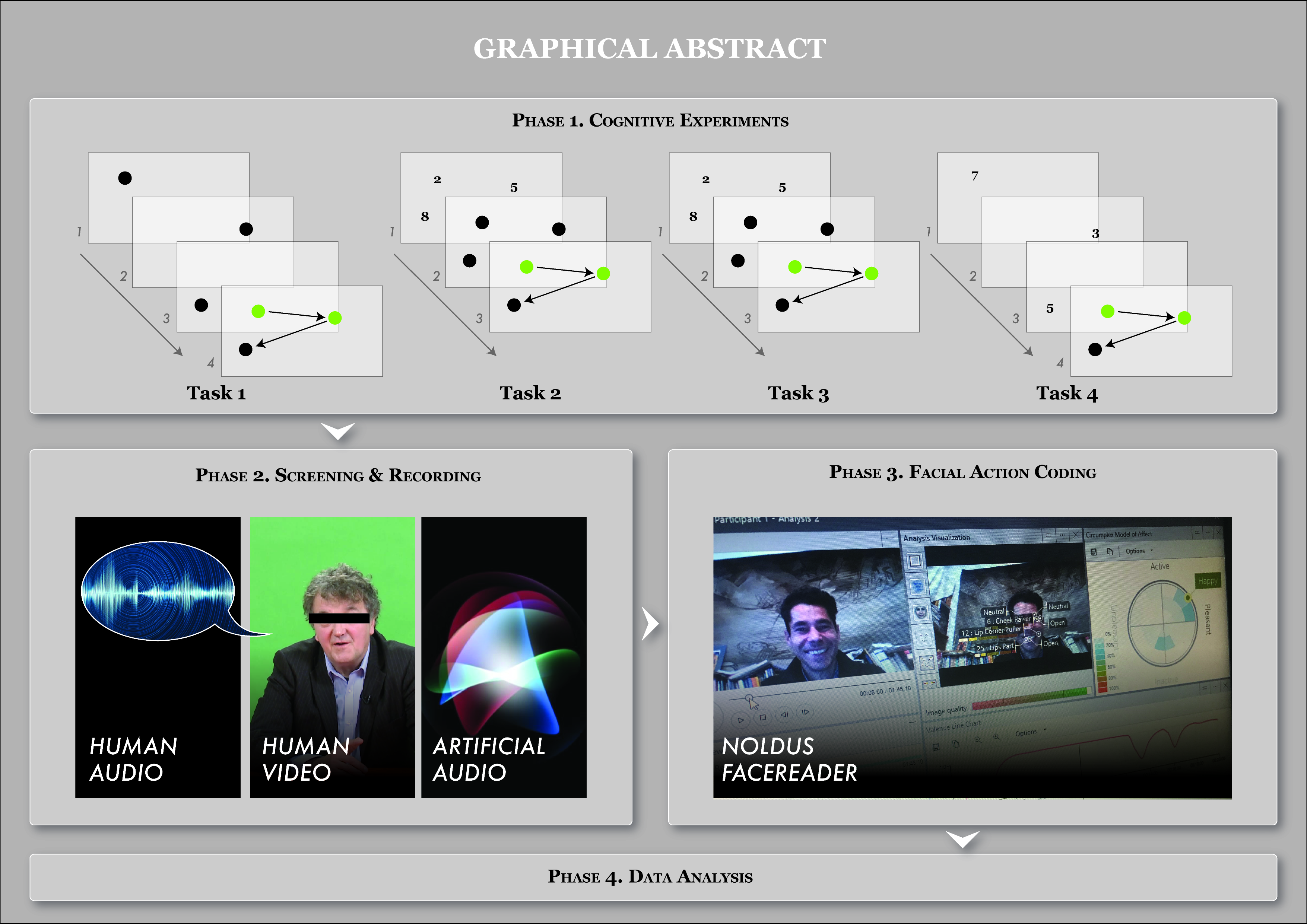

Supplement: Supplementary file 1 — Supplementary Material 1 [file 40359_2025_2590_MOESM1_ESM.tif]
