# Supplementary material for: Overconfident, but angry at least. AI-Based investigation of facial emotional expressions and self-assessment bias in human adults
Source: BMC Psychol. 2025 Mar 10;13:223. doi: 10.1186/s40359-025-02590-7 (PMC11895137; doi:10.1186/s40359-025-02590-7)
Supplement: Supplementary file 2 — Supplementary Material 2 [file 40359_2025_2590_MOESM2_ESM.docx]

**Appendix**

**GA**. Graphical Abstract shows the workflow of the data collection from the cognitive tests (Phase 1) through screening and recording (Phase 2) to facial action coding (Phase 3) that enabled data analysis (Phase 4).

**
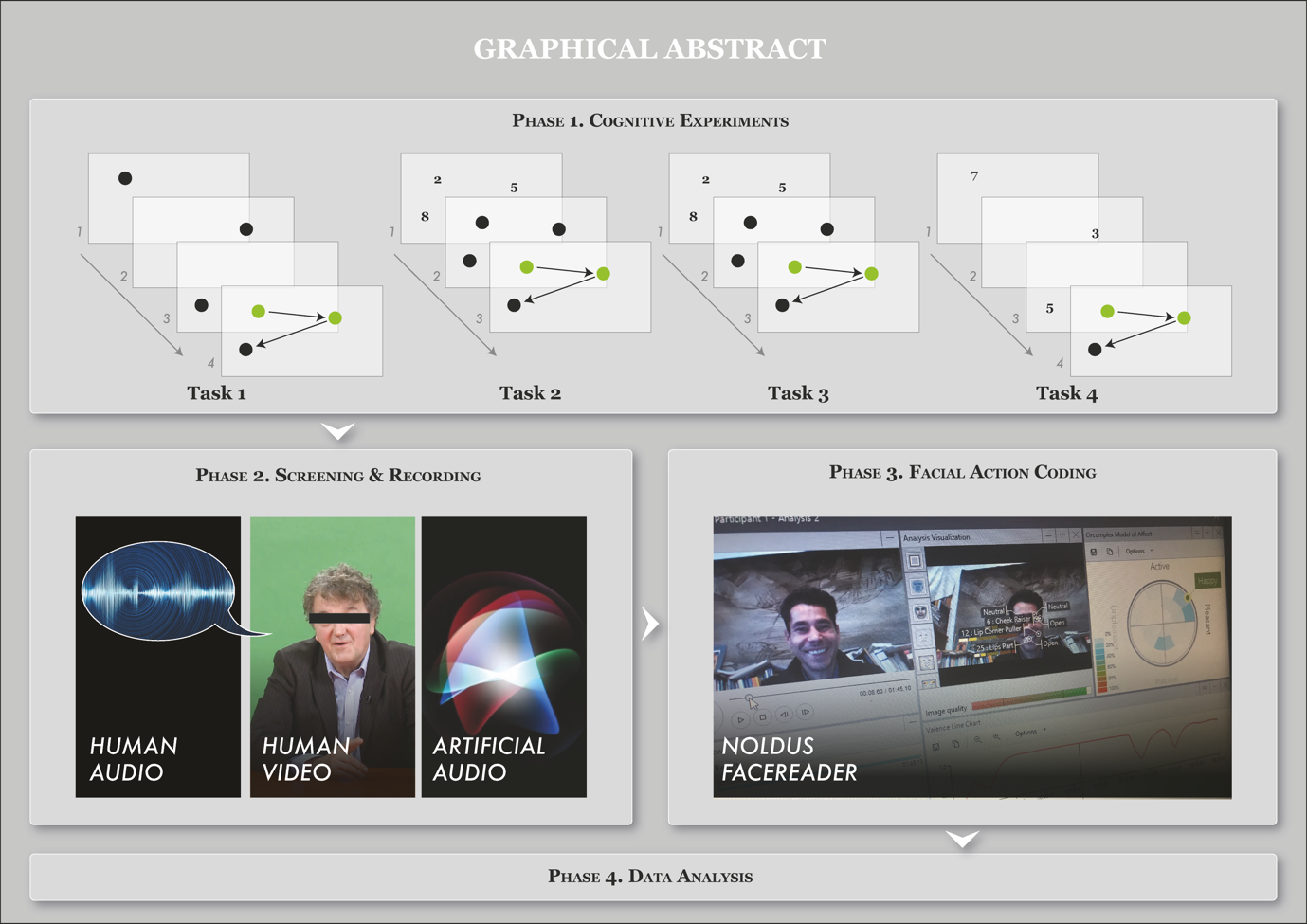
**

**Table 1A**. Dwass-Steel-Critchlow-Fligner pairwise comparisons confirm no evidence of differences between modalities – Human Audio (HA) and Video (HV), Artificial Audio (AA) – and their Average (A_E_) in the investigated Emotions, emotion Ratios – Saturation (SE) and Transparency (TE) and Personality categories.

|  |  | **HA-HV** | | **HA-AA** | | **HA-AE** | | **HV-AA** | | **HV-AE** | | **AA-AE** | |
| --- | --- | --- | --- | --- | --- | --- | --- | --- | --- | --- | --- | --- | --- |
|  |  | **W** | **p** | **W** | **p** | **W** | **p** | **W** | **p** | **W** | **p** | **W** | **p** |
| Emotions | Neutral | 0.9053 | 0.919 | -0.1246 | 1.000 | 0.0581 | 1.000 | -1.1213 | 0.858 | -0.9053 | 0.919 | 0.1661 | 0.999 |
|  | Happy | -0.3120 | 0.996 | 0.2100 | 0.999 | 0.8590 | 0.930 | 0.5980 | 0.975 | 1.1790 | 0.839 | 0.5100 | 0.984 |
|  | Surprised | -0.0759 | 1.000 | 1.2152 | 0.826 | 1.6449 | 0.650 | 1.2615 | 0.809 | 1.7112 | 0.621 | 0.3229 | 0.996 |
|  | Sad | 0.0090 | 1.000 | 0.4060 | 0.992 | 1.0691 | 0.874 | 0.1580 | 1.000 | 0.8393 | 0.934 | 0.6074 | 0.974 |
|  | Scared | 1.4142 | 0.750 | NaN | NaN | 1.4142 | 0.750 | -1.4142 | 0.750 | -0.0288 | 1.000 | 1.4142 | 0.750 |
|  | Disgusted | -0.1265 | 1.000 | -0.9281 | 0.914 | -0.0759 | 1.000 | -0.8189 | 0.938 | 0.0506 | 1.000 | 0.8735 | 0.926 |
|  | Angry | -1.3777 | 0.764 | -1.8402 | 0.562 | -0.0102 | 1.000 | -0.4851 | 0.986 | 1.6008 | 0.670 | 2.0665 | 0.461 |
|  | Other | -1.7110 | 0.621 | 1.6110 | 0.665 | 0.2240 | 0.999 | 2.9160 | 0.166 | 2.0680 | 0.461 | -1.4370 | 0.740 |
| Ratios | S_E_ | 0.6990 | 0.960 | -1.0400 | 0.883 | -0.2160 | 0.999 | -1.8710 | 0.548 | -1.2460 | 0.815 | 0.7230 | 0.957 |
|  | T_E_ | -0.0999 | 1.000 | 0.2580 | 0.998 | 0.1663 | 0.999 | 1.0394 | 0.883 | 0.3990 | 0.992 | -0.4237 | 0.991 |
| Personality | Extroversion | -0.3970 | 0.992 | 0.6850 | 0.963 | 1.1870 | 0.836 | 0.9190 | 0.916 | 1.6350 | 0.655 | 0.4910 | 0.986 |
|  | Neuroticism | 0.2055 | 0.999 | 0.4060 | 0.992 | 1.0690 | 0.874 | -0.0437 | 1.000 | 0.6118 | 0.973 | 0.6245 | 0.971 |
|  | Hostility | -1.4050 | 0.753 | -1.8450 | 0.560 | -0.3590 | 0.994 | -0.4140 | 0.991 | 1.2700 | 0.806 | 1.6590 | 0.644 |
| *Note. * p < .05, ** p < .01, *** p < .001* | | | | | | | | | | | | | |
